# Microplastics Exacerbate Cadmium-Induced Hepatotoxicity via the IRE1α/TXNIP/NLRP3 Axis-Driven Endoplasmic Reticulum Stress and Pyroptosis

**DOI:** 10.3390/vetsci13080748

**Published:** 2026-07-28

**Authors:** Yuxue Yang, Tong Guo, Haoran Deng, Xiaoyi Li, Fuhao Chen, Liyuan Huang, Shan Chen, Jiarui He, Jiangwei Xiang, Haocheng Huang, Hongchuan Deng, Kun Zhang, Zhijun Zhong, Ziyao Zhou, Guangneng Peng, Dechun Chen, Xu Song, Haifeng Liu

**Affiliations:** 1 College of Veterinary Medicine, Sichuan Agricultural University, Chengdu 611130, China; yyx02358@163.com (Y.Y.); guotong772025@163.com (T.G.); yqjm33@163.com (H.D.); vetchenfh@163.com (F.C.); hly4599@126.com (L.H.); 18382350916@163.com (H.H.); 18784998479@163.com (H.D.); 15281297124@163.com (K.Z.); zhongzhijun488@126.com (Z.Z.); zzhou@sicau.edu.cn (Z.Z.); pgn.sicau@163.com (G.P.); 2 Chongqing Fengdu Agricultural Science and Technology Development Group Co., Ltd., Chongqing 408200, China; 3 College of Animal and Veterinary Sciences, Southwest Minzu University, Chengdu 610041, China; chendechun@swun.edu.cn

**Keywords:** microplastics, cadmium, hepatotoxicity, endoplasmic reticulum stress, pyroptosis, animal model

## Abstract

**Background:** Microplastics (MPs) and cadmium (Cd) are widespread environmental pollutants posing significant health risks, but their combined hepatotoxic effects and underlying mechanisms remain poorly understood. **Methods**: Using in vivo and in vitro co-exposure models, we systematically investigated the impact of MPs on Cd-induced hepatotoxicity. The study assessed hepatic injury, oxidative stress, and inflammatory responses through transcriptomic analysis, histopathological examination, immunofluorescence, and quantitative real-time PCR. Pharmacological inhibition of endoplasmic reticulum stress with 4-phenylbutyric acid and selective blockade of IRE1α with MKC3946 were employed to dissect the signaling pathway. **Results**: Co-exposure to MPs and Cd significantly aggravated Cd-induced hepatic pathological damage, inflammation, and oxidative stress. Transcriptomic profiling revealed marked activation of the endoplasmic reticulum stress pathway and upregulation of pyroptosis-associated genes. Mechanistically, endoplasmic reticulum stress triggered pyroptosis via the IRE1α/TXNIP/NLRP3 signaling axis. Notably, inhibition of endoplasmic reticulum stress with 4-phenylbutyric acid, or selective blockade of IRE1α with MKC3946, effectively attenuated TXNIP/NLRP3 activation and the downstream pyroptotic response. **Conclusions**: MPs intensify Cd-induced hepatotoxicity by activating the IRE1α/TXNIP/NLRP3 pathway, leading to endoplasmic reticulum stress-driven pyroptosis. These findings provide a mechanistic framework for understanding the combined toxicity of microplastics and heavy metals, with important implications for environmental health risk assessment in animals and humans.

## Simple Summary

Microplastics are tiny plastic particles widely present in the environment. They often coexist with heavy metals like cadmium, which is a common environmental pollutant known to cause liver damage. This study investigated whether and how microplastics worsen cadmium-induced liver injury in mice and in liver cells. We found that exposure to both pollutants together caused more severe liver damage, inflammation, and oxidative stress than exposure to either pollutant alone. At the molecular level, microplastics exacerbated cadmium-induced endoplasmic reticulum stress and triggered a specific form of inflammatory cell death called pyroptosis via the IRE1α/TXNIP/NLRP3 signaling pathway. Importantly, inhibiting this pathway significantly reduced liver cell damage. These findings reveal that microplastics are not merely passive carriers but active toxicological enhancers of heavy metal toxicity, providing a crucial mechanistic framework for environmental health risk assessment in animals and humans. Our results highlight the importance of evaluating pollutant mixtures rather than single chemicals, informing future veterinary toxicological studies and the development of protective strategies for animal liver health under multi-pollutant environmental conditions.

## 1. Introduction

Microplastics (MPs) are a class of emerging environmental pollutants with widespread distribution. Their sources include the physical and chemical degradation of industrial plastics, as well as direct release from cleaning products, paints, cosmetics, and medical materials [1]. Extensive research has confirmed the ubiquity of MPs in water, soil, and air, leading to inevitable human and animal exposure through routine activities [2,3,4]. Once ingested, MPs are known to disrupt gut microbiota homeostasis, neurological function, and reproductive health [5,6,7], and have also been associated with neurotoxic effects and oxidative stress in various experimental models [8]. Importantly, due to their high surface area and lipophilic properties, MPs can act as carriers for heavy metals such as cadmium (Cd), thereby enhancing the bioavailability and toxic effects of these pollutants [9]. Polystyrene is one of the most commonly detected polymer types in environmental and biota samples, and is widely used as a model material in MPs toxicological studies due to its well-characterized physicochemical properties and commercial availability in uniform size fractions.

Cd is a well-recognized highly toxic environmental heavy metal. It is difficult for both humans and animals to metabolize or eliminate once ingested. Cd accumulates in various organs, including the liver, kidneys, heart, and bones, and is known to damage the reproductive and nervous systems, leading to multisystemic injury [10,11,12,13,14]. The liver, as the primary detoxification and metabolic organ, is a major target of Cd toxicity. Previous studies have shown that Cd exposure can lead to non-alcoholic fatty liver disease, alcoholic liver disease, liver fibrosis, and even hepatocellular carcinoma [15,16,17,18]. Although the molecular mechanisms of Cd-induced hepatotoxicity have been partially elucidated, growing evidence indicates that its toxic effects are significantly amplified by co-exposure to MPs [19]. Moreover, both MPs and Cd have been shown to induce endoplasmic reticulum stress (ERS) in various tissues [20,21,22]. Given that domestic and farm animals are increasingly exposed to environmental MPs and Cd through contaminated feed and water, understanding their combined hepatotoxic effects is of particular veterinary relevance.

ERS is a critical adaptive response to adverse microenvironments, such as nutrient deprivation, hypoxia, and oxidative stress [23,24]. It is widely recognized as a key factor in cellular dysfunction and pathological conditions. ERS is closely linked to the onset and progression of various liver diseases, including metabolic dysregulation, fibrosis, inflammation, and tumorigenesis [25]. ER dysfunction leads to the accumulation of misfolded or unfolded proteins in its lumen [26], disrupting intracellular calcium homeostasis, exacerbating oxidative damage, activating inflammatory responses, and ultimately undermining cellular stability and survival [27].

Upon ERS activation, cells initiate the unfolded protein response (UPR) to restore homeostasis [28]. The UPR is primarily mediated by three ER sensors: IRE1α, PERK, and ATF6, with the IRE1α–XBP1 pathway being the most evolutionarily conserved and functionally diverse. This pathway regulates protein quality control and influences immune responses, inflammation, and autophagy [29]. Recent studies have shown that the IRE1α signaling pathway plays a crucial role in regulating inflammation, pyroptosis, and oxidative stress [30]. Pyroptosis is a form of programmed cell death characterized by cell swelling, membrane pore formation, and the release of pro-inflammatory cytokines such as IL-1β and IL-18 [31]. Unlike other types of cell death, pyroptosis is driven by inflammasome activation, serving as a key link between cellular stress and inflammatory damage [32]. The NLRP3 inflammasome is considered a critical bridge between cellular stress and inflammation, and its activation not only promotes inflammatory responses but also exacerbates cellular and tissue injury [33].

TXNIP is a sensitive molecular marker of oxidative stress, regulated by ERS-associated genes [34]. Upon ERS activation, signaling pathways such as IRE1α–XBP1, PERK–eIF2α, and ATF6 upregulate TXNIP expression [35]. TXNIP enhances reactive oxygen species (ROS) generation by inhibiting the antioxidant thioredoxin (TRX) system, and directly binds to and activates the NLRP3 inflammasome, initiating pyroptosis [36]. Specifically, TXNIP activation not only increases ROS production but also alters its interaction with TRX through phosphorylation, relieving TRX inhibition and accelerating NLRP3 inflammasome activation.

Notably, increasing evidence indicates that ERS and pyroptosis are interconnected and act as key drivers of pathological processes [37,38,39]. Sustained activation of IRE1α further exacerbates the ERS response through downstream events, including ROS generation and TXNIP phosphorylation and upregulation, ultimately promoting pyroptosis [40]. Studies have demonstrated that the IRE1α signaling pathway plays a crucial role in oxidative stress and pyroptosis by upregulating TXNIP, thereby relieving TXNIP’s inhibition of the TRX system and promoting NLRP3 inflammasome activation [41].

Although existing studies have shown that both MPs and Cd can induce ERS [42,43], systematic research on their combined effects leading to overactivation of ERS and subsequent liver damage remains scarce. Most current research focuses on the individual effects of MPs or Cd on ERS, whereas the mechanisms underlying their combined action in inducing ERS and liver injury have not been thoroughly explored. This highlights a critical gap in scientific understanding that requires further investigation. We therefore hypothesized that MPs enhance Cd-induced hepatotoxicity by amplifying ERS through the IRE1α/TXNIP/NLRP3 signaling axis, thereby promoting pyroptosis.

With this contextual foundation, the present study investigates how co-exposure to MPs exacerbates Cd-induced hepatotoxicity at the mammalian level. Using both in vivo and in vitro models, we explored the integrated ERS–pyroptosis signaling axis. Our findings provide novel molecular insights into the hepatotoxic mechanisms underlying MPs–heavy metal co-pollution and offer essential theoretical foundations for evaluating its potential risks to livestock, companion animals, and wildlife health.

## 2. Materials and Methods

### 2.1. Animal Studies

A total of 72 SPF-grade male Kunming mice were used weighing between 35–40 g from Chengdu Dashuo Experimental Animal Co., Ltd. (Sichuan, China). Before the experiment began, the breeding area was disinfected with 75% alcohol wipes and ultraviolet light. During the entire experimental period, the mice were allowed free access to food and water. The feeding environment was room temperature (25 ± 5 °C). After one week of acclimatization, the mice were randomly divided into six groups of 12 mice each: control, Cd (4.8 mg/kg/d CdCl_2_), MPs low dose (1 mg/d MPs), MPs high dose (10 mg/d MPs), low-dose MPs + Cd (1 mg/d MPs + 4.8 mg/kg/d CdCl_2_), and high-dose MPs + Cd (10 mg/d MPs + 4.8 mg/kg/d CdCl_2_) [44,45,46,47]. The sample size (n = 12 per group) was determined based on our previous studies and preliminary experiments, which confirmed that this number was sufficient to detect statistically significant differences while accounting for potential attrition during the 6-week gavage period and allowing for multiple parallel assays (histology, biochemistry, qPCR, and transcriptomics). The CdCl_2_ solution and MPs were prepared using distilled water to achieve the desired concentrations and were administered via oral gavage. The mice were maintained under a 12 h light/12 h dark cycle, with gavage procedures conducted daily between 9:00 and 10:00 AM. Cage cleaning and bedding replacement occurred every other day. Mice were weighed weekly, and the gavage volume was adjusted based on body weight. The control group received an equivalent volume of distilled water over a 6-week period. Initial body weights were recorded, and final body weights were measured following an overnight fast from food and water prior to sampling. The weight growth rate was calculated using the formula: [(final weight − initial weight)/initial weight] × 100%. On the 43rd day of the experiment, blood samples were obtained from the mice via orbital extraction. The collected blood was then centrifuged to obtain the supernatant, which was subsequently stored at −80 °C for future analysis. Following blood collection, the mice were sacrificed by cervical dislocation, and the livers were excised for morphological analysis.

### 2.2. Scanning Electron Microscopy Observation of PS-MPs

MPs were selected for this test in vivo and in vitro. One milliliter of PS-MPs suspension was placed into a 1.5 mL centrifuge tube; the centrifuge was pre-cooled at 4 °C, the solution was centrifuge at 10,000 r/min for 20 min, and the supernatant was discarded to obtain the PS-MPs precipitate. The precipitate was placed in an oven at 60–70 °C to evaporate the water to obtain the dried PS-MPs powder. The samples were observed using a scanning electron microscope (Olympus, Tokyo, Japan).

### 2.3. Hematoxylin and Eosin (H&E) Staining of Liver

Fresh liver tissues were fixed in 4% paraformaldehyde (PFA) for 24 h at room temperature. After fixation, tissues were dehydrated through a graded ethanol series (70%, 80%, 90%, 95%, and 100%), cleared in xylene, and infiltrated with paraffin at 60 °C for 2 h before embedding in paraffin blocks. The paraffin-embedded tissues were then cut into 5 μm thick sections. Sections were placed into xylene, rehydrated through gradient concentrations of ethanol, and stained with hematoxylin and eosin (H&E) solutions. After staining, sections were dehydrated through graded ethanol, cleared in xylene, and sealed with neutral gum. Finally, the sections were observed under a light microscope (Olympus, Tokyo, Japan).

### 2.4. Biochemical Index Determination

Mouse serum samples were assayed for aspartate aminotransferase (AST) and alanine aminotransferase (ALT) using AST (Cat. no.: C010-2-1) and ALT (Cat. no.: C009-2-1) kits from the Nanjing Jiancheng Bioengineering Institute (Nanjing, China). AST and ALT levels were measured according to manufacturer’s instructions. The optical density (OD) of each well was measured using a microplate reader (Thermo Fisher Scientific, Waltham, MA, USA) at 510 nm.

Mouse serum samples were assayed for catalase (CAT) and superoxide dismutase (T-SOD) using CAT (Cat. no.: A007-1-1) and T-SOD (Cat. no.: A001-1) kits from the Nanjing Jiancheng Bioengineering Institute (Nanjing, China). CAT and T-SOD levels were measured according to manufacturer’s instructions. The optical density (OD) of each well was measured using a microplate reader (Thermo Fisher Scientific, USA) at 405 nm (CAT) and 550 nm (T-SOD).

### 2.5. ELISA Assay

Mouse liver tissue homogenate was assayed for IL-1β and IL-18 using IL-1β (KX2169) and IL-18 (KX2040) ELISA kits from Kexing Biopharm Co., Ltd. (Shanghai, China). IL-1β and IL-18 levels were measured according to manufacturer’s instructions. The optical density (OD) of each well was measured using a microplate reader (Thermo Fisher Scientific, USA) at 450 nm.

### 2.6. CCK-8

AML-12 cells were obtained from Shanghai Jinyuan Biotechnology Co., Ltd. (Shanghai, China). Cells were cultured in High Sugar DMEM Medium (G4511-500ML, Servicebio, Wuhan, China) at 37 °C. The medium was supplemented with 1% 100 μg/mL of penicillin and streptomycin (G4003-100ML, Servicebio). The specific operation was as follows: 12 h after the cells were uniformly inoculated into the 96-well plate, the cell attachment was observed under a microscope. The culture medium was aspirated, gently washed with PBS twice, and then replaced by the culture medium with the configured concentration. After 24 h of modeling, the medium was aspirated. CCK-8 was diluted with complete medium according to the instructions and added to the well plates; plates were incubated at 37 °C in the cell culture incubator to avoid light for 0.5–1 h. The OD value at 450 nm was detected using a microplate reader (Thermo Fisher Scientific, USA), and cell viability was calculated.

### 2.7. Cell Culture and Treatment

CdCl_2_, MPs, MKC3946, and 4-PBA were solubilized in High Sugar DMEM Medium. CdCl_2_, MPs, MKC3946, and 4-PBA were used to treat AML-12 cells for 24 h. The groups were treated as follows: control, Cd (20 μM CdCl_2_), MPs low dose (10 μg/mL MPs), MPs high dose (100 μg/mL MPs), low-dose MPs + Cd (10 μg/mL MPs + 20 μM CdCl_2_), high-dose MPs + Cd (100 μg/mL MPs + 20 μM CdCl_2_), MKC3946 (10 μM MKC3946), MPs + Cd + MKC3946 (100 μg/mL MPs + 20 μM CdCl_2_ + 10 μM MKC3946), 4-PBA (4 mM 4-PBA), and MPs + Cd + 4-PBA (100 μg/mL MPs + 20 μM CdCl_2_ + 4 mM 4-PBA).

### 2.8. ER Tracker Assay

According to the manufacturer’s instructions, the ER tracker (ER tracker-blue, MKBio, Shanghai, China, # MX4351) was used for ER localization. To investigate the expression level of NLRP3 and TXNIP in cells, cells were seeded on glass slides, fixed with methanol for 10 min. The staining working solution was diluted and pre-warmed according to the instructions, and the pre-warmed working solution was added and incubated for 15–30 min at 37 °C, protected from light. The slide was then inverted and placed on the glass slide that had been dipped in the anti-fluorescence quenching agent. Then, cells were observed using a laser confocal microscope (Olympus, Tokyo, Japan).

### 2.9. RNA Isolation and Quantitative Real-Time PCR

Total RNA was extracted from mouse liver tissue, AML-12, and their co-cultured cell systems using TRIzol reagent (Thermo Scientific, CAS: 593-84-0), and quantified by a NanoDrop1000 spectrophotometer (Thermo Scientific, Wilmington, DE, USA). RNA was then reverse transcribed using a universal reverse transcription kit (Beijing Solarbio Science & Technology Co., Ltd., Beijing, China: RP1105). The real-time PCR was then analyzed using a SYBR Green qPCR kit Novoprotein Scientific Inc., Suzhou, China) and primers. The primer sequences (Sangon Biotech, Shanghai, China) are listed in Table 1.

### 2.10. Omics Analysis

The difference analysis software is DESeq2, and the screening threshold of differentially expressed genes (DEGs) is |log2FC| ≥ 1 and *p*-value < 0.05. PLS-DA plots, expression heatmaps, and volcano plots were generated using the R software (v 4.2.0). Enrichment analysis was performed with Goatools software (v 3.0) using Fisher’s exact test. To control the calculated false positive rate, four multiple tests (Bonferroni, Holm, Sidak, and false discovery rate) were used to correct the *p*-value, and in general, if the corrected *p*-value (p_fdr) < 0.05, significant enrichment was considered to be present for that GO function.

### 2.11. Statistical Analyses

All experimental data were processed and statistically analyzed using GraphPad Prism 8.0 software. One-way ANOVA was performed for overall comparisons, followed by Tukey’s multiple comparisons test for pairwise post hoc analysis. A value of *p* < 0.05 was considered statistically significant. All experiments were conducted with at least three independent biological replicates, and results are expressed as mean ± SEM. For the Tukey’s post hoc test, group means were ranked in descending order and assigned lowercase letters (a, b, c, …), with the highest mean designated as “a.” Groups sharing the same letter are not significantly different (*p* > 0.05), whereas groups with different letters differ significantly (*p* < 0.05). To further evaluate the magnitude and precision of the observed effects, effect sizes (Cohen’s d) and 95% confidence intervals (CIs) were calculated for the key pairwise comparisons.

## 3. Results

### 3.1. Effects of MPs on Cd-Induced Subchronic Poisoning in Mice

**Figure 1:**
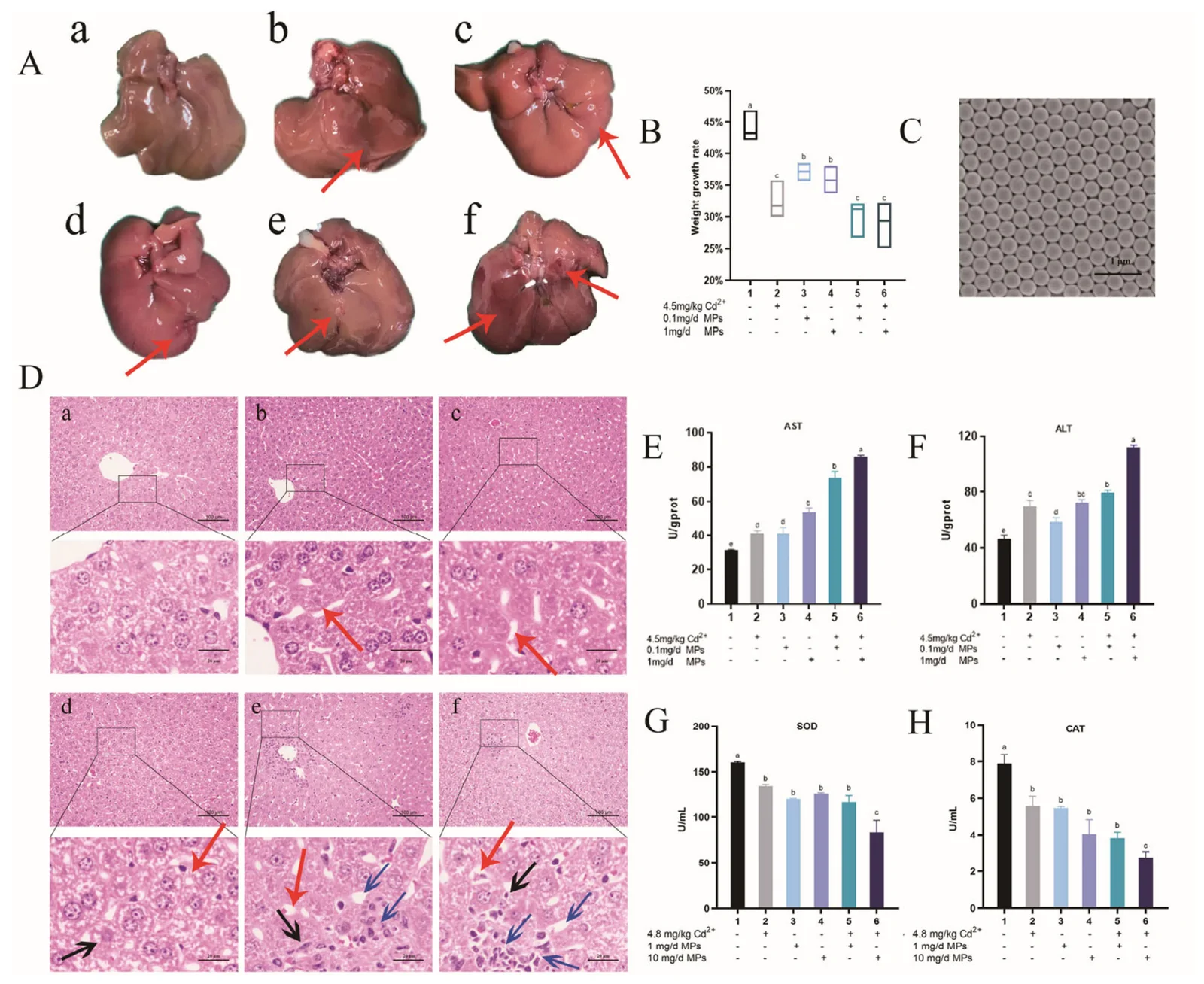
Weight growth rate, pathology, liver function and oxidative stress detection of mice livers. (**A**) Mice were intragastrically administered 4.8 mg/kg/d CdCl_2_ and 1 and 10 mg/d MPs for 6 weeks, then the livers morphology of mice in each group. In the figure, the red arrow indicates the visual pathological changes of the liver, and it can be observed that high-dose cadmium and microplastic combined poisoning results in significant hemorrhagic damage and bruising. Figures (**A**,**D**), (**a**,**b**,**c**,**d**,**e** and **f**) respectively, represent different groups, which are as follows: control, Cd (4.8 mg/kg/d CdCl_2_), MPs low dose (1 mg/d MPs), MPs high dose (10 mg/d MPs), low-dose MPs + Cd (1 mg/d MPs + 4.8 mg/kg/d CdCl_2_),and high-dose MPs + Cd (10 mg/d MPs + 4.8 mg/kg/d CdCl_2_). (**B**) Body weight growth rate of groups. (**C**) Scanning electron microscopy of PS-MPs. (**D**) The livers were extracted for HE staining (scale bar, 100 µm). Among them, the red arrow indicates the disorder of liver lobules and the swelling of cytoplasm; the black arrow indicates the destruction of cell membranes; the blue arrow indicates the dissolution of nuclei and vacuolar degeneration. The enzyme activities of AST (**E**) and ALT (**F**), SOD (**G**), CAT (**H**), in serum of mice were assessed through intragastric administration of 4.8 mg/kg/d CdCl_2_ and 1, 10 mg/d MPs for 6 weeks. There were significant differences between two or more letter groups (*p* < 0.05), and the same letter indicated no significant difference between different groups (*p* > 0.05).

Scanning electron microscopy analysis of polystyrene MPs with a particle size of 0.5 μm confirmed uniformity in particle size distribution (Figure 1C). Compared with the control group, the experimental groups demonstrated a significantly reduced growth rate in body weight (Figure 1B). Morphological evaluation of liver tissues across different groups revealed that those exposed to Cd in conjunction with MPs exhibited more severe lesions, characterized by hemorrhagic lesions and ecchymosis, as indicated by the red arrow in Figure 1A. The group exposed to low-dose MPs displayed liver morphology akin to the Cd group, with occasional disorganization of hepatic cords and cytoplasmic swelling, as indicated by the red arrow in Figure 1D(a–c). In contrast, the high-dose MPs group showed substantial pathological changes, including irregular arrangement of hepatic cords and partial disruption of cell membranes, as indicated by the red arrow in Figure 1D(d). In the cohorts subjected to concurrent exposure to Cd and MPs, there was a notable increase in granular and vacuolar degeneration, as well as localized infiltration of inflammatory cells, alongside extensive nucleolysis and disorganized cellular architecture, as indicated by the red arrow in Figure 1D(e,f). The enzymatic activities of alanine aminotransferase (ALT) and aspartate aminotransferase (AST) were significantly elevated in the groups exposed to combined toxicity, in contrast to those subjected to individual toxicants. Moreover, a pronounced differential increase in ALT and AST activities was observed in the high-dose MPs + Cd group compared with the low-dose MPs + Cd group (refer to Figure 1E,F). No significant differences were observed among the three groups exposed to single toxicants. Additionally, the activities of superoxide dismutase (SOD) and catalase (CAT) were significantly reduced in the high-dose MPs + Cd group (refer to Figure 1G,H).

### 3.2. Transcriptomic Analysis of the Effects of Cd and MPs on the Liver of the Mice

**Figure 2:**
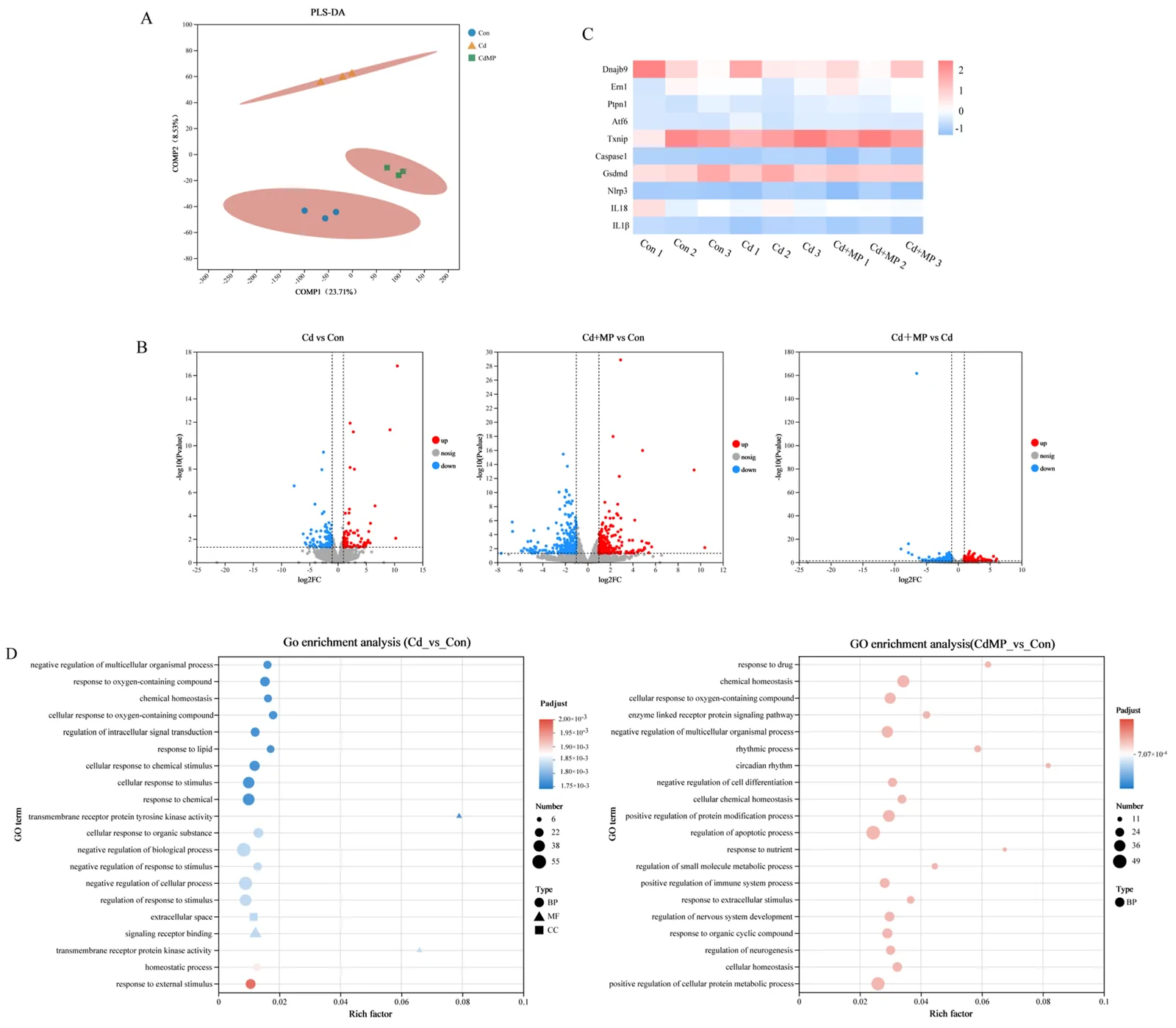
Omics analysis of mouse liver tissue. (**A**) PLS-DA score plots of liver tissues of the three groups of mice. (**B**) Heatmap of genes: red indicates a positive correlation, blue indicates a negative correlation, with color intensity reflecting statistical significance. (**C**) Volcano plot of differentially expressed genes in liver tissue. (**D**) KEGG pathway enrichment results.

To further clarify the specific mechanism of combined Cd and MPs toxicity on liver damage in mice, we performed transcriptomics sequencing on the control group, Cd group, and high-dose MPs + Cd group, and the PLS-DA showed that there were significant differences among the three groups and good parallelism within the groups (Figure 2A), and we defined genes that satisfied the criteria of |log2FC| > 1 and *p* < 0.05 as differentially expressed genes, and we could see that the Cd + MPs vs. Con group had more differentially expressed genes (ERS-related genes: TXNIP and ATF6; pyroptosis-related genes: Caspase-1, GSDMD, NLRP3, IL-1β, and IL-18) than the Cd vs. Con group (Figure 2B), and we found that genes related to ERS and pyroptosis were altered in the transcriptomic data (Figure 2C).

The differentially expressed genes screened in the previous experiments were pathway-enriched by the KEGG database, and *p* < 0.05 was used as the threshold for significant differential pathway screening. Compared with the control group, the liver tissue response to oxygen-containing compounds, the negative regulation of the multicellular organism process, the regulation of the apoptotic process, and other pathways were significantly altered in Cd-induced liver injury. Compared with the control group, the combined toxicity induced by Cd and MPs significantly altered the liver tissue response to oxygen-containing compounds, the regulation of the pyroptosis process, and the positive regulation of both protein metabolism and other pathways (Figure 2D).

### 3.3. Effect of MPs on Cd-Induced ERS In Vitro and In Vivo

**Figure 3:**
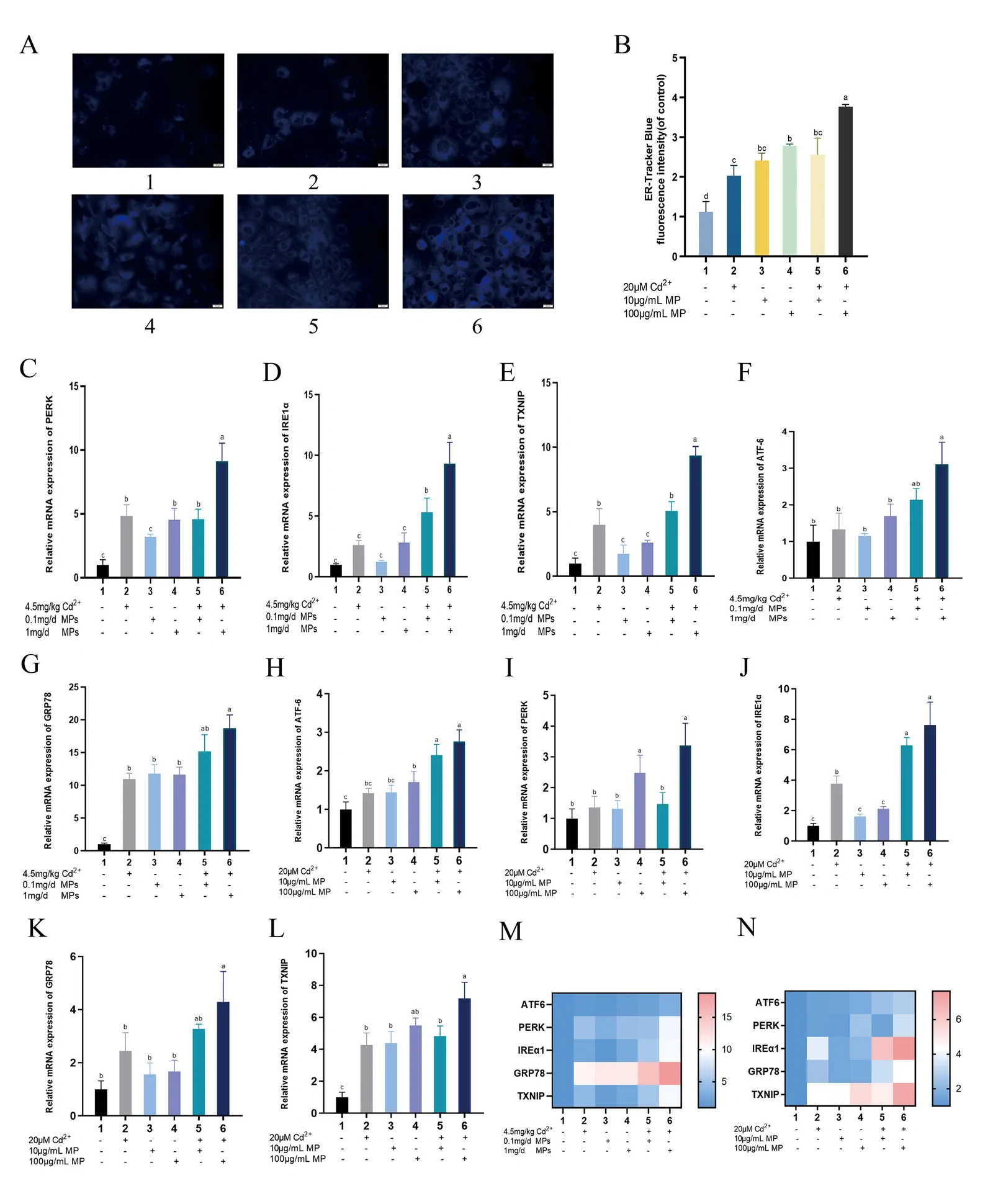
Research on ERS tracing and related marker proteins. (**A**,**B**) ER tracker-blue staining (scale bar, 20 µm). (**C**–**G**,**M**) Mice were intragastrically administered 4.8 mg/kg/d CdCl_2_ and 1, 10 mg/d MPs for 6 weeks. The qPCR was performed to analyze the ERS marker proteins GRP78, PERK, IRE1α, ATF6, and TXNIP in mice livers. (**H**–**L**,**N**) AML-12 cells were exposed to Cd at 20 μM and to MPs at 10 and 100 μg/mL for 24 h. The qPCR was performed to analyze the ERS marker proteins GRP78, PERK, IRE1α, ATF6, and TXNIP in AML-12 cells. (*p* < 0.05).

As illustrated in Figure 3A,B, the fluorescence intensity of the ER in AML-12 cells was markedly elevated in all experimental groups relative to the control group. Notably, the intensity was significantly higher in the high-dose MPs + Cd group compared with the Cd^2+^ (20 μM) group. This increased fluorescence intensity indicates more severe damage to the ER. As illustrated in Figure 3H–L, our observations indicate that, relative to the control group, the mRNA expression levels of GRP78, PERK, and TXNIP were significantly elevated in the Cd-treated group. In comparison to the Cd group, the addition of a low concentration of low-dose MPs combined with Cd did not result in a significant change in the relative mRNA expression of GRP78, ATF6, PERK, and TXNIP. However, the introduction of a high concentration of high-dose MPs combined with Cd led to a significant increase in the relative expression of GRP78, PERK, IRE1α, ATF6, and TXNIP, thereby demonstrating ERS characteristics in a dose-dependent manner. These findings are consistent with the observations made in mouse liver (Figure 3C–G).

### 3.4. Effect of MPs on Cd-Induced Pyroptosis In Vitro and In Vivo

**Figure 4:**
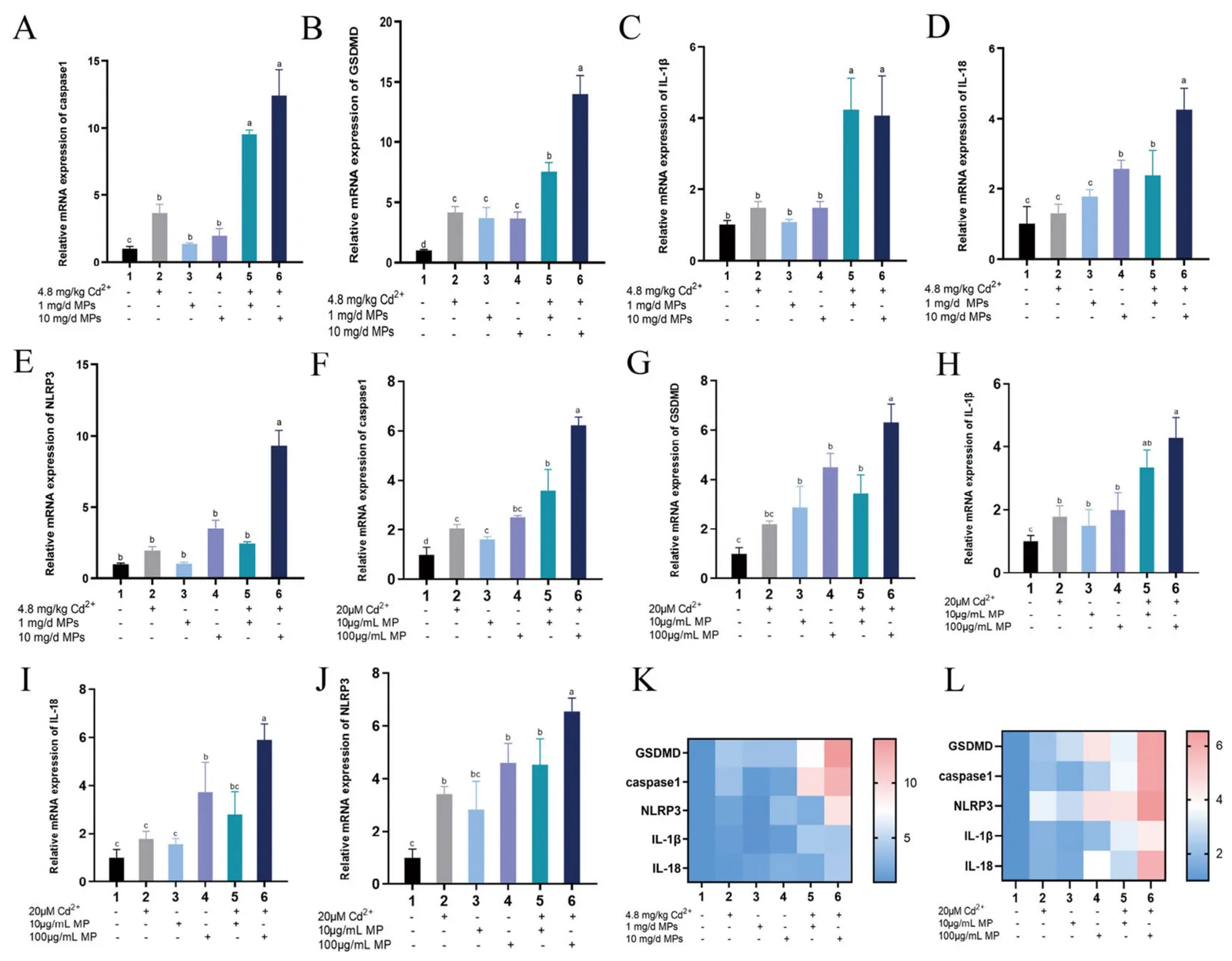
Research on pyroptosis-related marker proteins. (**A**–**E**,**K**) Mice were intragastrically administered 4.8 mg/kg/d CdCl_2_ and 1, 10 mg/d MPs for 6 weeks. qPCR was performed to analyze the pyroptosis marker proteins Caspase-1, GSDMD, IL-1β, IL-1,8 and NLRP3 in mice livers. (**F**–**J**,**L**) AML-12 cells were exposed to Cd^2+^ at 20 μM and to MPs at 10 and 100 μg/mL for 24 h. The qPCR was performed to analyze the ERS marker proteins Caspase-1, GSDMD, IL-1β, IL-18, and NLRP3 in AML-12 cells. There were significant differences between two or more letter groups (*p* < 0.05), and the same letter indicated no significant difference between different groups (*p* > 0.05).

As illustrated in Figure 4F–J, the relative mRNA expression levels of GSDMD and Caspase-1 were significantly elevated in the Cd group, while no significant changes were observed in the relative expression levels of NLRP3, IL-1β, and IL-18 when compared with the control group. In contrast, the low-dose MPs + Cd group exhibited significantly increased relative expression levels of GSDMD, Caspase-1, IL-1β, and IL-18, with no significant alteration in NLRP3 expression. Furthermore, in the high-dose MPs + Cd group, the relative expression levels of Caspase-1, GSDMD, IL-1β, IL-18, and NLRP3 all demonstrated a significant or highly significant increase in a dose-dependent manner. As depicted in Figure 4A–E, these observations are consistent with those observed in mouse liver.

### 3.5. Effect of the ERS–IRE1α/TXNIP Pathway on Pyroptosis in AML-12 Cells

**Figure 5:**
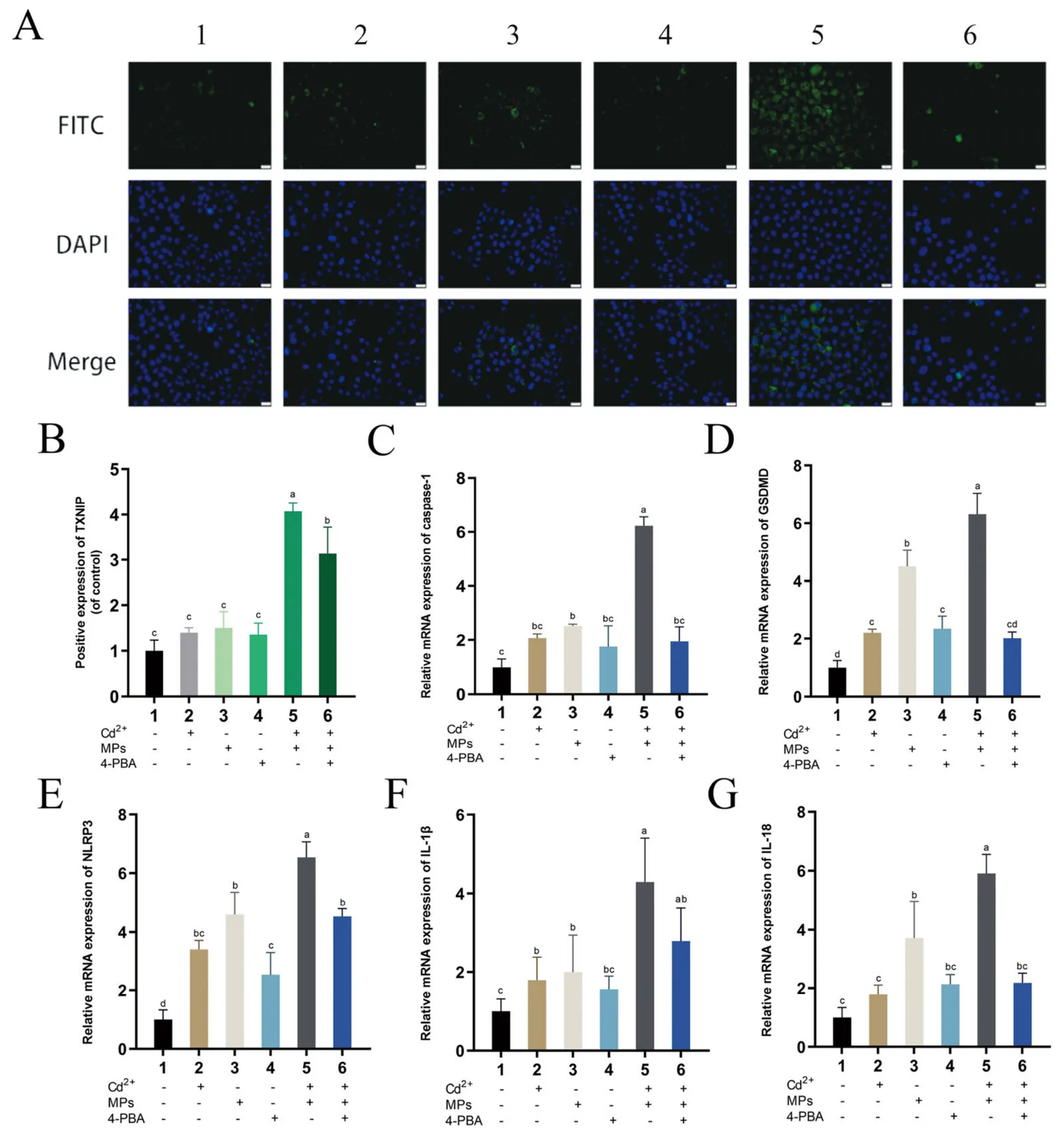
The effect of ERS inhibitor 4-PBA on pyroptosis in vitro. (**A**,**B**) TXNIP ER tracker-blue staining (scale bar, 20 µm). AML-12 cells were exposed to Cd at 20 μM, MPs at 10 and 100 μg/mL, and 4-PBA at 4 mM for 24 h. (**C**–**G**) AML-12 cells were exposed to Cd at 20 μM, MPs at 10 and 100 μg/mL, and 4-PBA at 4 mM for 24 h. qPCR was performed to analyze the pyroptosis marker proteins Caspase-1, GSDMD, NLRP3, IL-1β, and IL-18 in AML-12 cells. There were significant differences between two or more letter groups (*p* < 0.05), and the same letter indicated no significant difference between different groups (*p* > 0.05).

**Figure 6:**
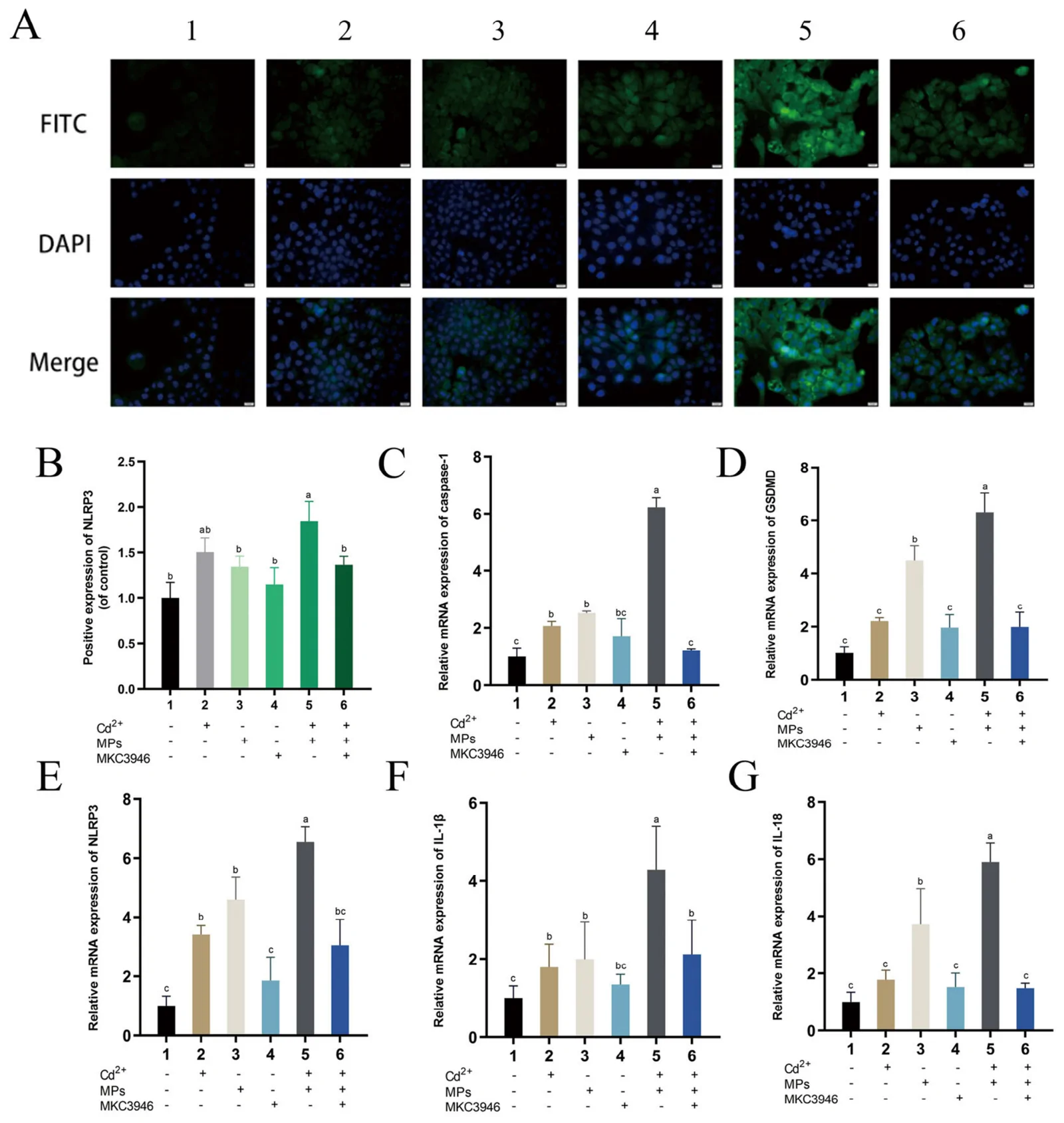
The effect of IRE1α inhibitor MKC3946 on pyroptosis in vitro. (**A**,**B**) NLRP3 ER tracker-blue staining (scale bar, 20 µm). AML-12 cells were exposed to Cd at 20 μM, to MPs at 10 and 100 μg/mL, and to MKC3946 at 4 mM for 24 h. (**C**–**G**) AML-12 cells were exposed to Cd at 20 μM, MPs at 10 and 100 μg/mL, and 4-PBA at 4 mM for 24 h. qPCR was performed to analyze the pyroptosis marker proteins Caspase-1, GSDMD, NLRP3, IL-1β, and IL-18 in AML-12 cells. There were significant differences between two or more letter groups (*p* < 0.05), and the same letter indicated no significant difference between different groups (*p* > 0.05).

During the 4-PBA inhibition phase, the application of 4-PBA led to a marked reduction in the fluorescent staining intensity of TXNIP compared with the Cd + MPs group (Figure 5A,B), suggesting an alleviation of ERS. This was further supported by a significant decrease in the relative mRNA expression levels of Caspase-1, GSDMD, NLRP3, IL-1β, and IL-18 (Figure 5C–G), indicating that TXNIP inhibition modulates NLRP3 expression and mitigates pyroptosis. Similarly, during the MKC3946 inhibition phase, the introduction of MKC3946 resulted in a substantial reduction in the fluorescent staining intensity of NLRP3 compared with the Cd + MPs group (Figure 6A,B), again suggesting an alleviation of ERS. Correspondingly, there was a significant decrease in the relative mRNA expression levels of GSDMD, Caspase-1, NLRP3, IL-1β, and IL-18 (Figure 6C–G), indicating that IRE1α inhibition influences NLRP3 expression.

## 4. Discussion

In this study, we systematically investigated the combined effects of MPs and Cd on liver injury using both in vivo and in vitro models. Our results demonstrate that co-exposure to MPs and Cd significantly exacerbates Cd-induced hepatotoxicity, as evidenced by more severe histopathological damage, elevated serum ALT/AST levels, and increased oxidative stress. Mechanistically, MPs amplify Cd-induced endoplasmic reticulum stress (ERS) and promote pyroptosis through the IRE1α/TXNIP/NLRP3 signaling axis. Pharmacological inhibition of ERS (with 4-PBA) or IRE1α (with MKC3946) effectively attenuated TXNIP/NLRP3 activation and downstream pyroptotic responses, confirming the causal role of this pathway.

The structural damage observed in mouse livers—hepatocyte swelling, disorganized hepatic cords, and inflammatory infiltration—was substantially more pronounced in the co-exposure groups than in single-exposure groups. Consistent with these morphological changes, serum ALT and AST levels were significantly elevated, indicating compromised hepatocyte membrane integrity and liver dysfunction [48]. Importantly, MPs enhanced Cd-induced hepatotoxicity in a dose-dependent manner. These findings align with previous reports that long-term MP exposure alone can induce liver inflammation and elevate transaminases [49,50], and that Cd exposure triggers inflammatory infiltration and oxidative stress [51]. Our data further show that co-exposure to MPs and Cd led to a marked reduction in SOD and CAT activities, two key antioxidant enzymes. This suggests that MPs exacerbate Cd-induced oxidative stress, an effect consistent with a previous study on combined MP and Cd exposure in aquatic organisms [52]. Similarly, combined exposures to different environmental pollutants have been shown to induce complex oxidative stress responses that differ from single exposures [53]. Together, these results indicate that MPs act as aggravating factors in Cd-induced oxidative liver damage.

Oxidative stress is a well-known trigger of ERS, a cellular response that aims to restore ER homeostasis through the unfolded protein response (UPR) [54,55,56,57]. However, when ERS is excessive or prolonged, it can switch from a protective to a detrimental role, ultimately leading to cell death [58,59,60]. In the present study, transcriptomic analysis revealed significant upregulation of ERS-related genes (e.g., TXNIP, ATF6) and pyroptosis-related genes (e.g., Caspase-1, GSDMD, NLRP3) in the co-exposure group, suggesting that aberrant activation of these pathways contributes critically to hepatocyte injury. In vitro, ER tracker staining showed intensified fluorescence in the high-dose MPs + Cd group, indicating aggravated ER damage. qPCR analyses further demonstrated dose-dependent increases in ERS markers (GRP78, PERK, IRE1α, ATF6, TXNIP) and pyroptosis markers (Caspase-1, GSDMD, IL-1β, IL-18, NLRP3) in both mouse liver and AML-12 cells. These data provide strong evidence that MPs amplify Cd-induced ERS and drive the subsequent pyroptotic response.

**Figure 7:**
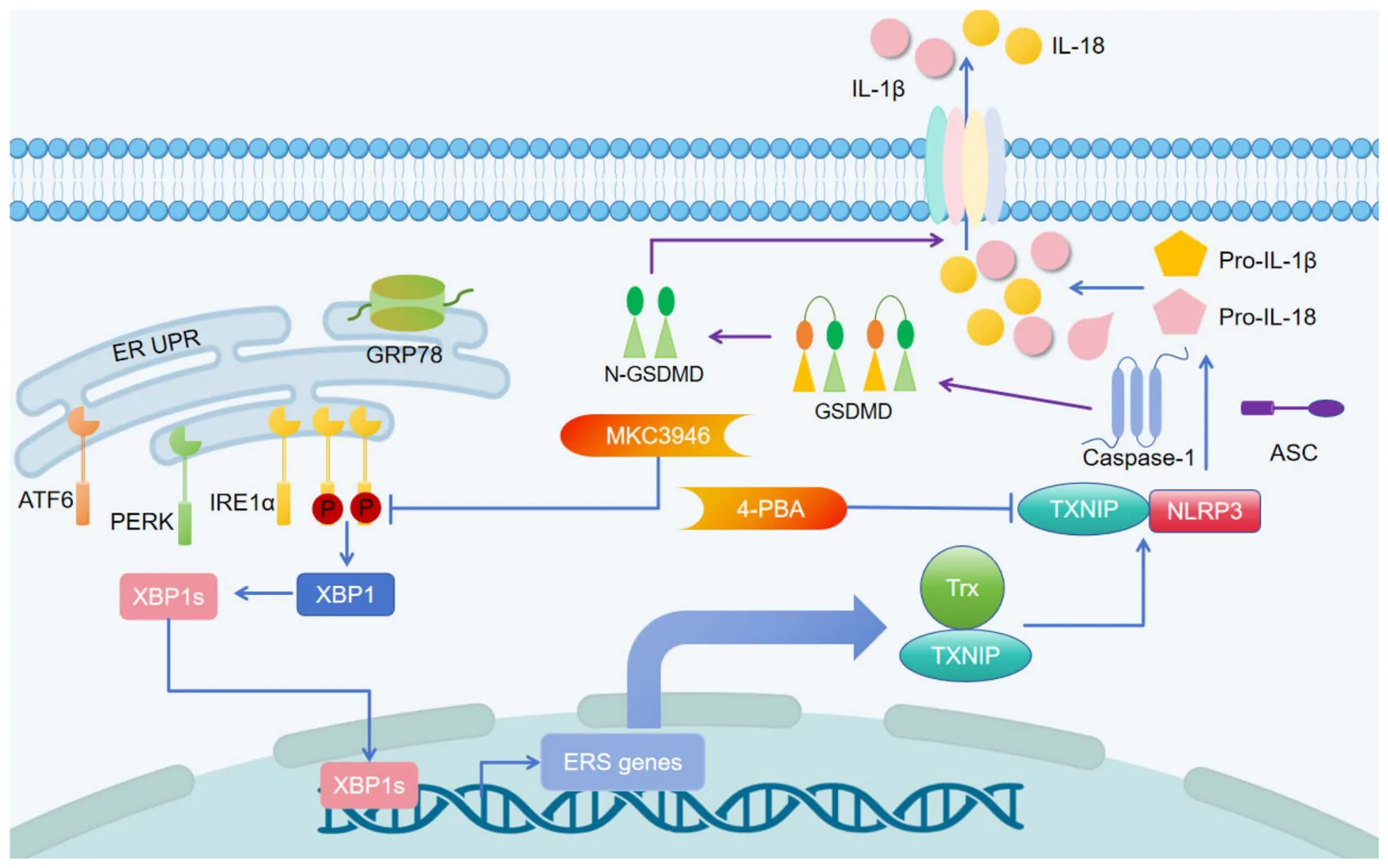
Schematic representation of cadmium-induced endoplasmic reticulum stress and cell pyroptosis in mouse liver.

**Figure 8:**
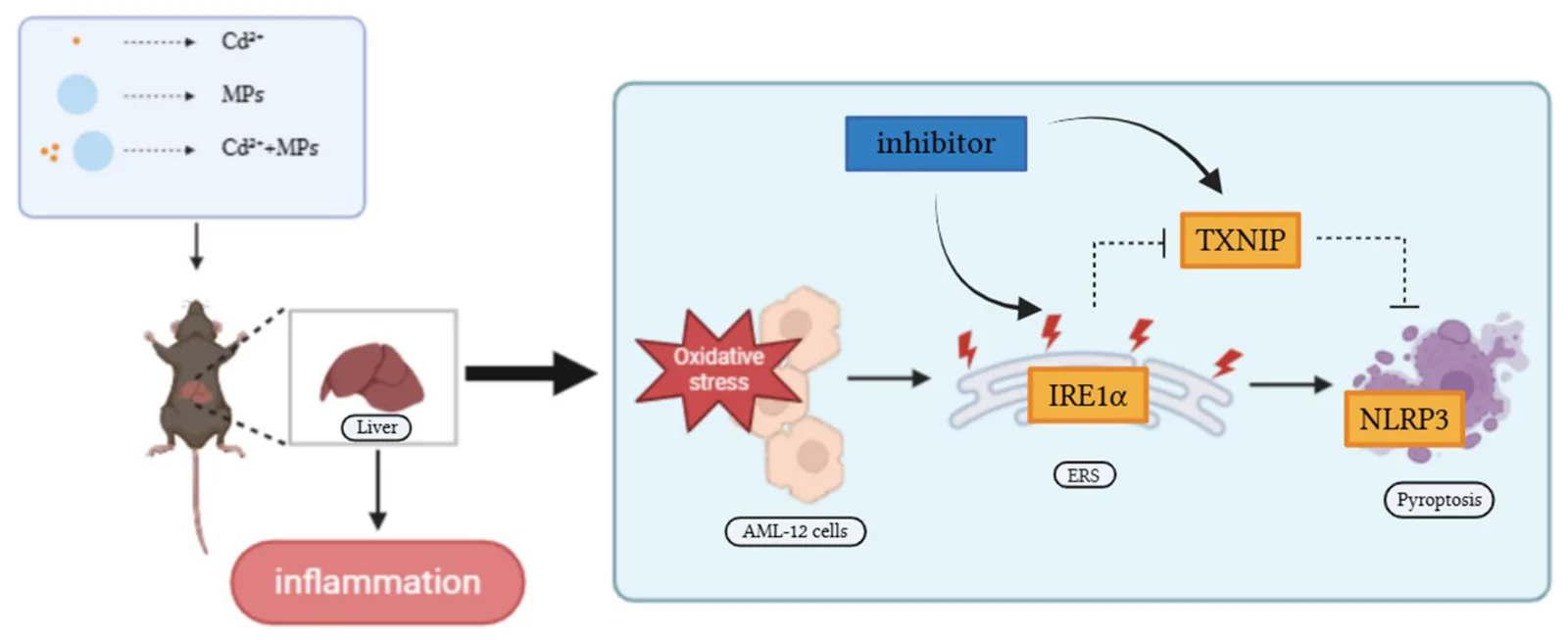
Graphical summary.

The NLRP3 inflammasome is a central regulator of pyroptosis and can be activated by various stress signals, including ERS [34,55]. TXNIP, a redox-sensitive protein, acts as a critical link between ERS and NLRP3 activation. Under conditions of oxidative stress, TXNIP is upregulated and binds to NLRP3, promoting inflammasome assembly and pyroptosis [61,62,63]. Our study shows that ERS inhibition with 4-PBA significantly reduced TXNIP expression, which in turn downregulated NLRP3, Caspase-1, GSDMD, IL-1β, and IL-18, demonstrating that ERS-mediated TXNIP upregulation is essential for NLRP3 inflammasome activation in this context. To identify the specific ERS sensor responsible, we targeted IRE1α, the most evolutionarily conserved branch of the UPR [64]. Pharmacological blockade of IRE1α with MKC3946 markedly reduced NLRP3 activation and subsequent pyroptotic gene expression, confirming that IRE1α is a key upstream regulator of the TXNIP/NLRP3 pathway under MPs + Cd exposure. These results are consistent with previous studies in other disease models where IRE1α regulated TXNIP/NLRP3-mediated pyroptosis [65,66,67]. Therefore, our study establishes a clear mechanistic cascade: MPs and Cd co-exposure → oxidative stress → ERS (via IRE1α) → TXNIP upregulation → NLRP3 inflammasome activation → pyroptosis → hepatocyte injury (summarized in Figure 7 and Figure 8).

Our findings extend current knowledge by revealing that MPs are not merely passive carriers of Cd but active modulators that amplify ER stress and pyroptotic cell death. This is particularly relevant for veterinary and environmental health, as domestic and farm animals are increasingly exposed to co-contaminated feed and water sources [9,19]. Compared with previous studies that focused on either MP- or Cd-induced ERS alone [20,21,22,42,43], our work provides an integrated mechanism linking the two. Additionally, while ERS has been implicated in Cd hepatotoxicity [18,68], we demonstrate that the presence of MPs triggers a more robust IRE1α-dependent pyroptosis pathway, suggesting a synergistic rather than additive effect.

Several limitations should be acknowledged. First, we used a single size (0.5 μm) and type (polystyrene) of MPs; however, environmental MPs exhibit wide heterogeneity in size, shape, polymer composition, and surface modifications. Whether other types of MPs produce similar aggravating effects remains to be determined. Second, this study focused on liver toxicity; MPs and Cd may also interact in other organs (e.g., kidney, intestine, nervous system) through distinct mechanisms. Third, the exposure duration (6 weeks) and doses, while effective for establishing a mechanistic model, may not fully represent long-term, low-dose environmental exposure scenarios. Future studies using environmentally relevant concentrations and longer exposure periods are warranted. Fourth, our oxidative stress assessment relied solely on SOD and CAT activities; the absence of ROS, MDA, and GSH/GSSG measurements prevents a comprehensive evaluation of the redox imbalance and downstream oxidative damage. Fifth, while our mechanistic findings are primarily based on qRT-PCR analyses at the mRNA level, we acknowledge that protein-level validation (e.g., Western blotting) would further strengthen the conclusions regarding ERS and pyroptosis pathway activation. Future studies should incorporate protein detection to complement the transcriptional data. Sixth, we did not measure Cd accumulation in liver tissue; thus, whether MPs exacerbate Cd toxicity by increasing Cd uptake or by amplifying downstream inflammatory signaling remains unresolved and warrants future investigation. Additionally, while we identified IRE1α as a critical mediator based on our transcriptomic screening and qPCR data showing its predominant activation, the potential involvement of other ERS sensors (PERK, ATF6) cannot be completely excluded and requires further investigation using specific pharmacological inhibitors or genetic approaches. 

From a veterinary perspective, these findings highlight the need for monitoring MPs and Cd co-exposure in livestock production systems, particularly in areas with high industrial pollution. Future studies should investigate whether nutritional interventions targeting the IRE1α/TXNIP/NLRP3 pathway could mitigate hepatotoxicity in farm animals.

In conclusion, this study provides compelling evidence that MPs significantly exacerbate Cd-induced hepatotoxicity by promoting ERS and pyroptosis via the IRE1α/TXNIP/NLRP3 signaling axis. Our findings reveal a synergistic interplay between microplastic pollution and heavy metal toxicity, highlighting that MPs act as active toxicological enhancers rather than inert vectors. These insights not only deepen the mechanistic understanding of co-pollutant hepatotoxicity but also identify IRE1α as a potential therapeutic target for mitigating combined chemical toxicity. Given the global rise in both microplastic and heavy metal contamination, this study underscores the importance of evaluating environmental risks from a multi-pollutant perspective and supports the development of targeted interventions to protect liver health in animals and humans.

## Figures and Tables

**Table 1:** Primer sequences used for qRT-PCR.

Name	Forward Primer	Reverse Primer
β-actin	CGTTGACATCCGTAAAGACCTC	TAGGAGCCAGGGCAGTAATCT
ATF6	AGCGCCCAAGACTCAAACC	CTGTATGCTGATAATCGACTGCT
GRP78	TCGATACTGGCCGAGACAAC	CGACGGTTCTGGTCTCACAC
PERK	CAGTGGGATTTGGACGTGGG	GAAGTTTTGTGGGTGCCCTCTG
IRE1α	CCCAAATGTGATCCGCTACT	TTGAGAGAATGCAGGTGTGC
TXNIP	AGTGATTGGCAGCAGGTC	GGTGTCTGGGATGTTTAGG
GSDMD	AGTGCTCCAGAACCAGAACCG	TCTGCCCTGAATGTTCCCATC
NLRP3	CAAGGCTGCTATCTGGAGGAA	TGCAACGGACACTCGTCATC
Caspase1	GCATGCCGTGGAGAGAAACA	TGGGCCTTCTTAATGCCATCAT
IL-1β	TGCCACCTTTTGACAGTGATG	TGATGTGCTGCTGCGAGATT
IL-18	TCCAACTGCAGACTGGCAC	GGCAGGAGTCCAGAAAGCAT

## Data Availability

The original contributions presented in this study are included in the article. Further inquiries can be directed to the corresponding authors.
